# Absent in melanoma 2 attenuates proliferation and migration and promotes apoptosis of human colorectal cancer cells by activating P38MAPK signaling pathway

**DOI:** 10.32604/or.2023.042986

**Published:** 2023-12-28

**Authors:** ZHI ZHANG, XIAOSONG LI, YING ZHANG, HAO ZHU, ZHENGUO QIAO, YANG LU, XIUWEI MI, HUIHUA CAO, GENHAI SHEN, SONGBING HE

**Affiliations:** 1 Department of General Surgery, Suzhou Ninth People’s Hospital, Suzhou Ninth Hospital Affiliated to Soochow University, Suzhou, China; 2 Medical College of Nantong University, Nantong, China; 3 Department of Gastroenterology, Suzhou Ninth People’s Hospital, Suzhou Ninth Hospital Affiliated to Soochow University, Suzhou, China; 4 Department of General Surgery, The First Affiliated Hospital of Soochow University, Suzhou, China; 5 Department of Oncological Surgery, Kunshan Traditional Hospital Affiliated to Nanjing University of Chinese Medicine, Kunshan, China

**Keywords:** Absent in melanoma 2, Proliferation, Migration, Apoptosis, P38MAPK, Colorectal cancer

## Abstract

Colorectal cancer (CRC) stands among the top prevalent cancers worldwide and holds a prominent position as a major contributor to cancer-related mortality globally. Absent in melanoma 2 (AIM2), a constituent of the interferon-inducible hematopoietic interferon-inducible nuclear antigens with 200 amino acid repeats protein family, contributes to both cancer progression and inflammasome activation. Despite this understanding, the precise biological functions and molecular mechanisms governed by AIM2 in CRC remain elusive. Consequently, this study endeavors to assess AIM2’s expression levels, explore its potential antitumor effects, elucidate associated cancer-related processes, and decipher the underlying signaling pathways in CRC. Our findings showed a reduced AIM2 expression in most CRC cell lines. Elevation of AIM2 levels suppressed CRC cell proliferation and migration, altered cell cycle by inhibiting G1/S transition, and induced cell apoptosis. Further research uncovered the participation of P38 mitogen-activated protein kinase (P38MAPK) in AIM2-mediated modulation of CRC cell apoptosis and proliferation. Altogether, our achievements distinctly underscored AIM2’s antitumor role in CRC. AIM2 overexpression inhibited proliferation and migration and induced apoptosis of CRC cells via activating P38MAPK signaling pathway, indicating AIM2 as a prospective and novel therapeutic target for CRC.

## Introduction

Colorectal cancer (CRC) stands among the top prevalent cancers, characterized by distressingly high incidence and mortality. This cancer type contributes to approximately 10% of total annual cancer diagnoses and cancer-related deaths globally [1–3]. CRC ranks third (10.0%) in incidence and second (9.4%) in mortality, with >1.9 million new cases and 935,000 deaths worldwide in 2020 [4]. Despite the advancement of endoscopy, surgery, radiotherapy, chemotherapy, biologics, and systemic therapy, which have led to improved clinical outcomes, the mortality rate of CRC remains high, especially for individuals diagnosed at advanced or metastatic stages [1,5,6]. Thus, it is essential to delve into the potential molecular mechanisms driving CRC progression, identify novel biomarkers for early detection, and pinpoint therapeutic targets for CRC.

Absent in melanoma 2 (AIM2), a novel identified inflammatory body, has garnered escalating attention due to its crucial involvement in cancer development. AIM2, as a constituent of the interferon-inducible hematopoietic interferon-inducible nuclear antigens with 200 amino acid repeats protein family, possesses the capability to bind to cytosolic double-stranded deoxyribonucleic acid (DNA). This binding stimulates the formation of the AIM2-inflammasome complex and activates caspase-1. This activation, subsequently causes the release of interleukin (IL)-1β and IL-18, thereby initiating the innate immune response [7–9].

Notably, recent research underscores AIM2’s dual role in cancer progression and inflammasome activation, highlighting its influence on the advancement and evolution of tumors [10]. While AIM2 level is reduced in breast cancer, prostate cancer, and hepatocellular carcinoma, it is overexpressed in nasopharyngeal carcinoma, cervical cancer, oral squamous cell carcinoma, and non-small cell lung cancer [11–17]. Significantly, our previous study affirms mitigated AIM2 level in CRC and establishes its absence as closely linked to unfavorable outcomes in CRC. This positions AIM2 as a potential independent and substantial prognostic factor for the survival of CRC patients [6]. However, scant studies delved into unraveling AIM2’s biological functions in CRC. The precise mechanisms underlying AIM2’s exact functional roles and molecular mechanisms in CRC’s development and progression remain ambiguous.

This study first reaffirmed the reduced AMI2 level in CRC cells and explored its impact on CRC cell cycle, apoptosis, proliferation, and migration. We then focused on unveiling the potential molecular mechanisms governing AIM2’s influence on CRC’s biological functions. Given the powerful potential of bioinformatics analysis to identify biomarkers and illuminate tumorigenesis on the molecular plane [18–20], we employed gene set enrichment analysis (GSEA) in tandem with our RNA sequencing (RNA-Seq) data and the Kyoto Encyclopedia of Genes and Genomes (KEGG) database. Our investigation uncovers significant correlations between AIM2 expression and cancer-related processes and signaling pathways, thereby reinforcing AIM2’s involvement in CRC progression, particularly through P38 mitogen-activated protein kinase (P38MAPK). Thus, our findings illuminate AIM2’s role in CRC progression and the underlying molecular machinery.

## Materials and Methods

### Cell culture

Six human CRC cell lines (HT29, SW620, SW480, HCT8, HCT116, and RKO) from the Cell Bank of the Chinese Academy of Sciences (Shanghai, China) were routinely maintained in high-glucose Dulbecco’s Modified Eagle Medium (DMEM; Gibco, Grand Island, NY, USA) with 5% penicillin/streptomycin (Gibco) and 10% fetal bovine serum (Gibco) in a humidified incubator with 5% CO_2_ at 37°C.

### Western blot

Proteins were isolated from CRC cell lines and underwent Western blot analysis, as documented previously [6]. The primary antibodies included anti-B-cell lymphoma-2 (Bcl-2), anti-Bcl-2 Associated X Protein (Bax), anti-protein 53 (P53), anti-AIM2, anti-P38MAPK, and anti-p-P38MAPK from Cell Signaling Technology (Beverly, MA, USA), as well as anti-β-tubulin and anti-β-actin from Proteintech (Rosemont, IL, USA). Ultimately, protein levels were assessed using ImageJ software.

### Lentivirus transfection and construction of stable cell line

AIM2 overexpression (OE) and negative control (NC) lentivirus vectors were from Genechem Co. (Shanghai, China) and used to transduced HCT116 cells, complying with the manufacturer’s instruction. In detail, 5 × 10^4^ cells per well were cultivated in six-well dishes. At 24 h of culture, lentivirus and HitransG P were introduced into the culture based on the multiplicity of infection. Following transduction, the medium was changed after 12–16 h. At 72 h, the green fluorescent protein (GFP) level was assessed under a fluorescence microscope to calculate the proportion of GFP-expressing cells. To establish an AIM2-overexpressing stable cell line, cells showing GFP expression were further selectively propagated by culturing them in a medium with 4 µg/mL of puromycin (Meilunbio, Dalian, China) for 10–14 days.

### Cell proliferation assay

Cell proliferation was inspected using the Cell Counting Kit-8 (CCK8; Dojindo, Shanghai, China), complying with the manufacturer’s instruction. 4,000 CRC cells/well were inoculated into 96-well plates. After 0, 24, 48, 72, and 96 h, cells were reacted with 10 µL of the CCK-8 reagent for 1–4 h, and OD_450 nm_ was assessed using a microplate reader (Molecular Devices, San Jose, CA, USA).

### Colony formation assay

400 CRC cells per well in a 96-well plate were cultivated for approximately 14 days, with medium refreshing per 3–4 days. After fixed in 4% paraformaldehyde at room temperature (RT) for 30 min, cells were stained with 0.1% crystal violet for 10 min at RT. The images were taken using a high-definition smartphone (Huawei, Shenzhen, China), and the number of colonies with >100 cells was assessed at 40× magnification under a light microscope.

### Wound healing assay

2 × 10^6^ CRC cells per well in six-well plates were cultivated to 80%–90% confluence. A vertical wound was created by gently scratching cells in the middle using a 200-μL pipette tip. After washed with phosphate-buffered saline (PBS), cells were maintained in serum-free medium for 0, 24, and 48 h, and the progress of wound closure was closely monitored and photographed.

### Cell cycle analysis

Cell cycle progression was assessed using a Cell Cycle Detection Kit (MultiSciences, Hangzhou, China) following manufacturer’s description. In short, CRC cells in six-well plates were harvested, washed with PBS, treated with 10 μL of permeabilization solution and 1 mL of DNA staining solution for 30 min in the dark at RT and subjected to flow cytometry (BD Biosciences, San Jose, CA, USA). Cells at various cell cycle phases were assessed using FlowJo software (Version 10.6.2).

### Apoptosis analysis

Cell apoptosis was investigated using the allophycocyanin (APC) Annexin V Apoptosis Detection Kit with propidium iodide (PI; MultiSciences, Hangzhou, China), following manufacturer’s description. In short, CRC cells cultivated in six-well plates were harvested, washed twice with pre-cooled PBS solution, and redispersed in 500 μL of binding buffer. The samples were double-stained with 10 μL of PI and 5 μL of Annexin V-APC in the dark at RT for 5 min. Apoptotic cells was captured using flow cytometry (BD Biosciences, San Jose, CA, USA) and assessed using FlowJo software (Version 10.6.2).

### Bioinformatics analysis

AIM2 is known to be critical in CRC progression. To uncover differentially expressed genes (DEGs) between AIM2 OE and NC groups, we outsourced RNA-Seq of HCT116 cells in the two groups and subsequent bioinformatic analysis to BGI Genomics Co., Ltd. (Shenzhen, China). To predict the possible signaling pathways and correlated functions of these DEGs influenced by AIM2 expression in CRC, we performed GSEA analysis according to our RNA-Seq data and the KEGG database.

### Statistics

All experiments were independently conducted in triplicate. Statistical analyses were executed using SPSS 26.0 (IBM, Armonk, NY, USA). Data were shown as means ± SD. Significant disparities between any two groups were examined using the *t*-test. *p* < 0.05 was set as significant.

## Results

### AIM2 level and overexpression efficiency in human CRC cells

**Figure 1:**
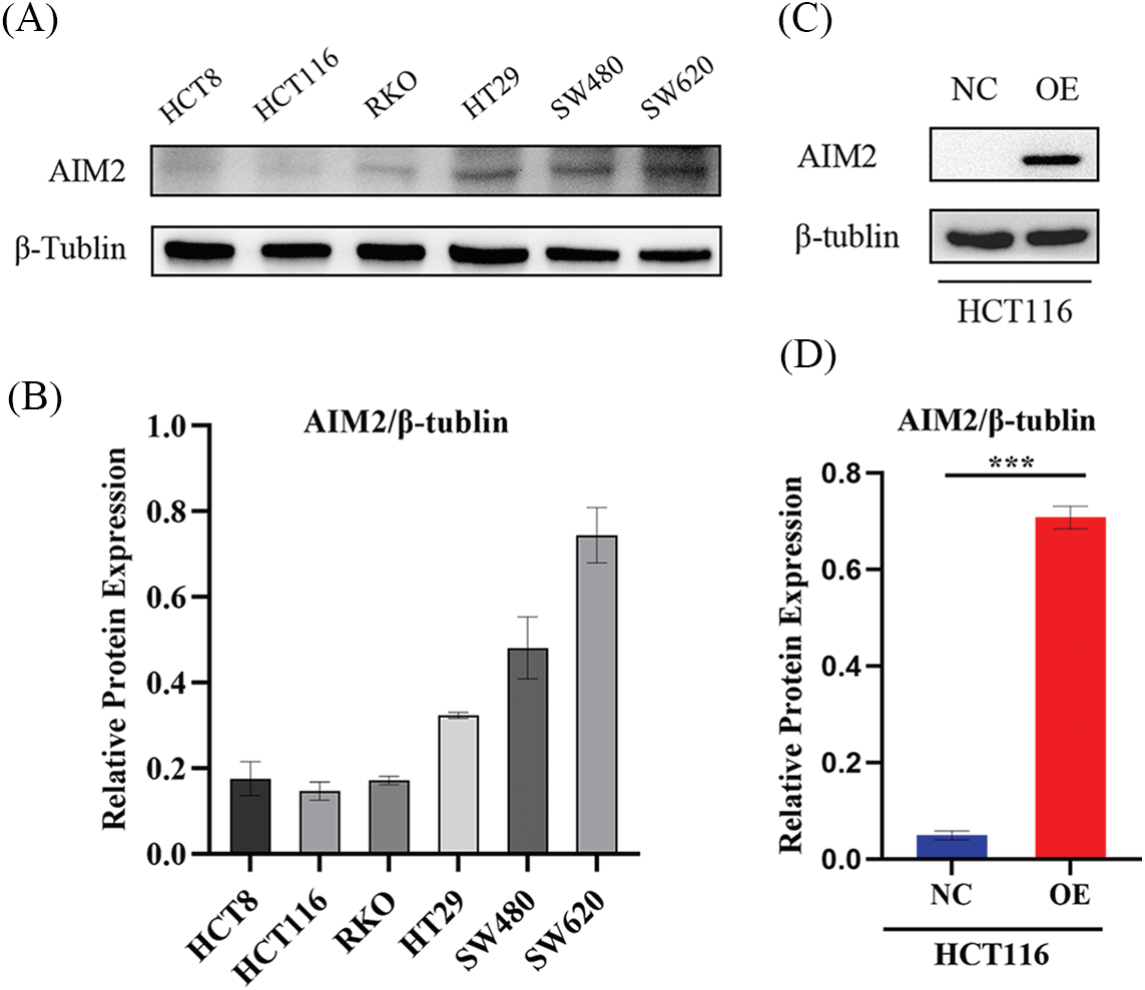
AIM2 expression and overexpression efficiency in human CRC cells. (A, B) Western blotting analysis of AIM2 protein expression in six different cell lines. Protein quantification results are shown. Error bars represent SD (n = 3). (C, D) Western blotting shows AIM2 protein levels in HCT116 cells stably transfected with NC or OE. Error bars represent SD (n = 3). NC: negative control; OE: AIM2 overexpression. ***p < 0.001.

Our previous studies [6] have revealed the prevalent low AIM2 levels in various CRC cell lines, with downregulation in human CRC tissues correlating with adverse clinical outcomes. To further explore AIM2’s biological functions in CRC cells and investigate its potential role in CRC development, we re-assessed AIM2 expression in HCT8, HCT116, RKO, HT29, SW480, and SW620 using Western blot. The results underscored the relatively lower AIM2 expression in these cell lines, with HCT116 cells showing the lowest AIM2 expression (Figs. 1A and 1B). This prompted us to select HCT116 cells for further studies. For subsequent experiments, AIM2 OE and NC lentivirus vectors were transduced into HCT116 cells, respectively. AIM2 OE efficacy was validated using Western blot. As shown in Figs. 1C and 1D, AIM2 expression was markedly increased by transducing the AIM2 OE lentiviral vector compared to cells transduced with the NC lentivirus vector (*p* < 0.001).

### AIM2 hinders CRC cell proliferation and migration

**Figure 2:**
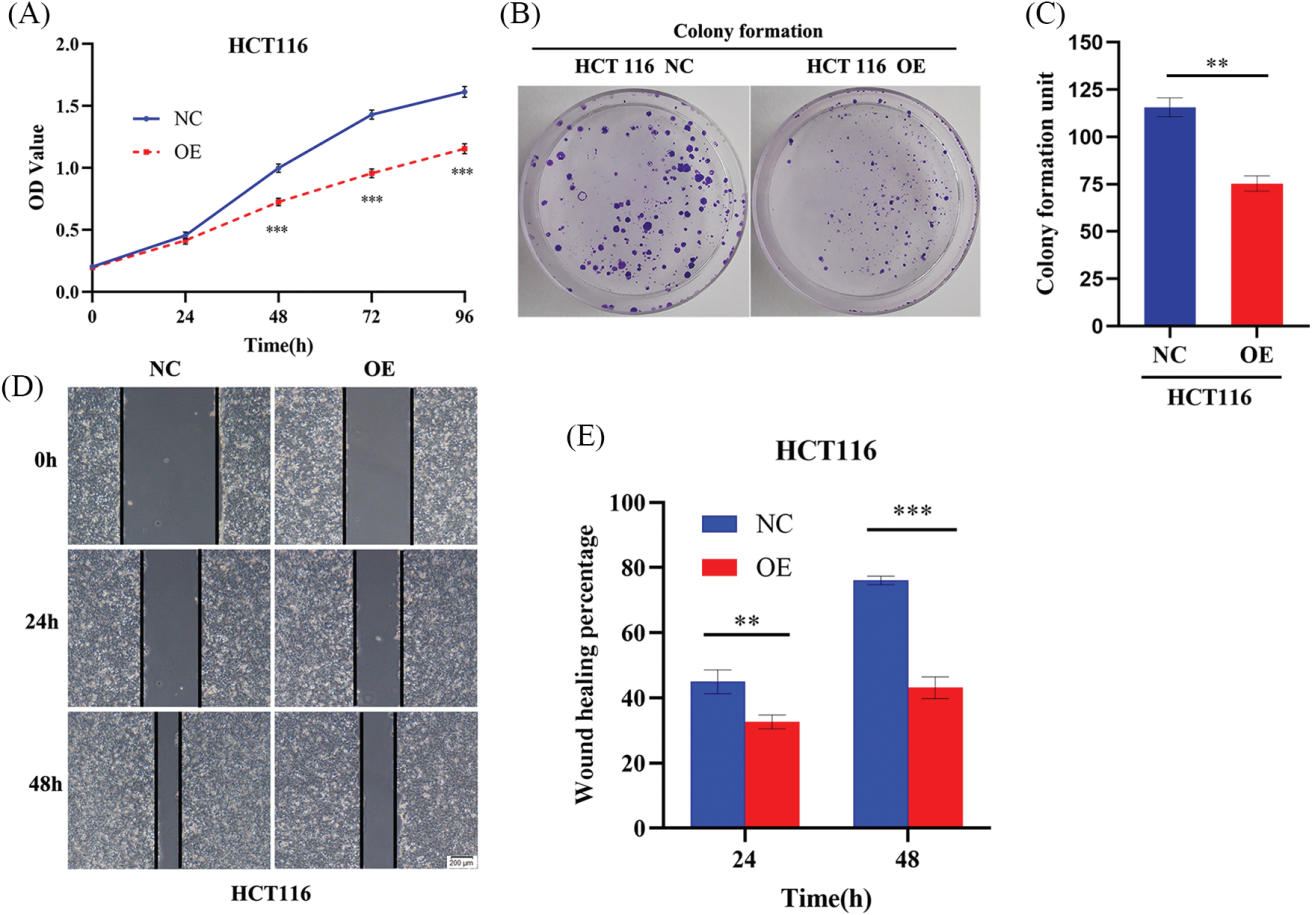
AIM2 hinders CRC cell proliferation and migration. (A) CCK8 assay-derived growth curves of HCT116 cells in the NC and OE groups. (B, C) Colony formation assays of HCT116 cells transfected with NC or OE. (D, E) Wound healing assays undertaken to assess AIM2’s effect on HCT116 cell migration at 24 and 48 h in the NC and OE groups. Error bars represent SD (n = 3). NC: negative control; OE: AIM2 overexpression. **p < 0.01, ***p < 0.001.

We have previously found that AIM2 level was commonly attenuated in CRC tissues and cell lines, with poor prognostic implications for CRC patients [6]. Consequently, we sought to detect whether AIM2 is responsible for regulating CRC cell phenotype and biological behavior. CCK8 assays revealed a noteworthy reduction in CRC cell proliferation rate in the AIM2 OE group in comparison to the NC group (*p* < 0.001, Fig. 2A). To further elucidate AIM2’s influence on cell proliferation, colony formation experiments were employed. The results concurred with the CCK8 findings, as the OE group exhibited significantly fewer colonies and reduced colony formation ability than the NC group (*p* < 0.01, Figs. 2B and 2C). These results substantiate our assumption that AIM2 repressed CRC cell proliferation. Subsequent wound healing assay indicated that cells migrated into wounded areas more slowly in the OE group compared to the NC group at 24 and 48 h (*p* < 0.001, Figs. 2D and 2E), underscoring AIM2’s inhibitory effect on CRC migration.

### AIM2 arrests CRC cell cycle and prompts apoptosis

**Figure 3:**
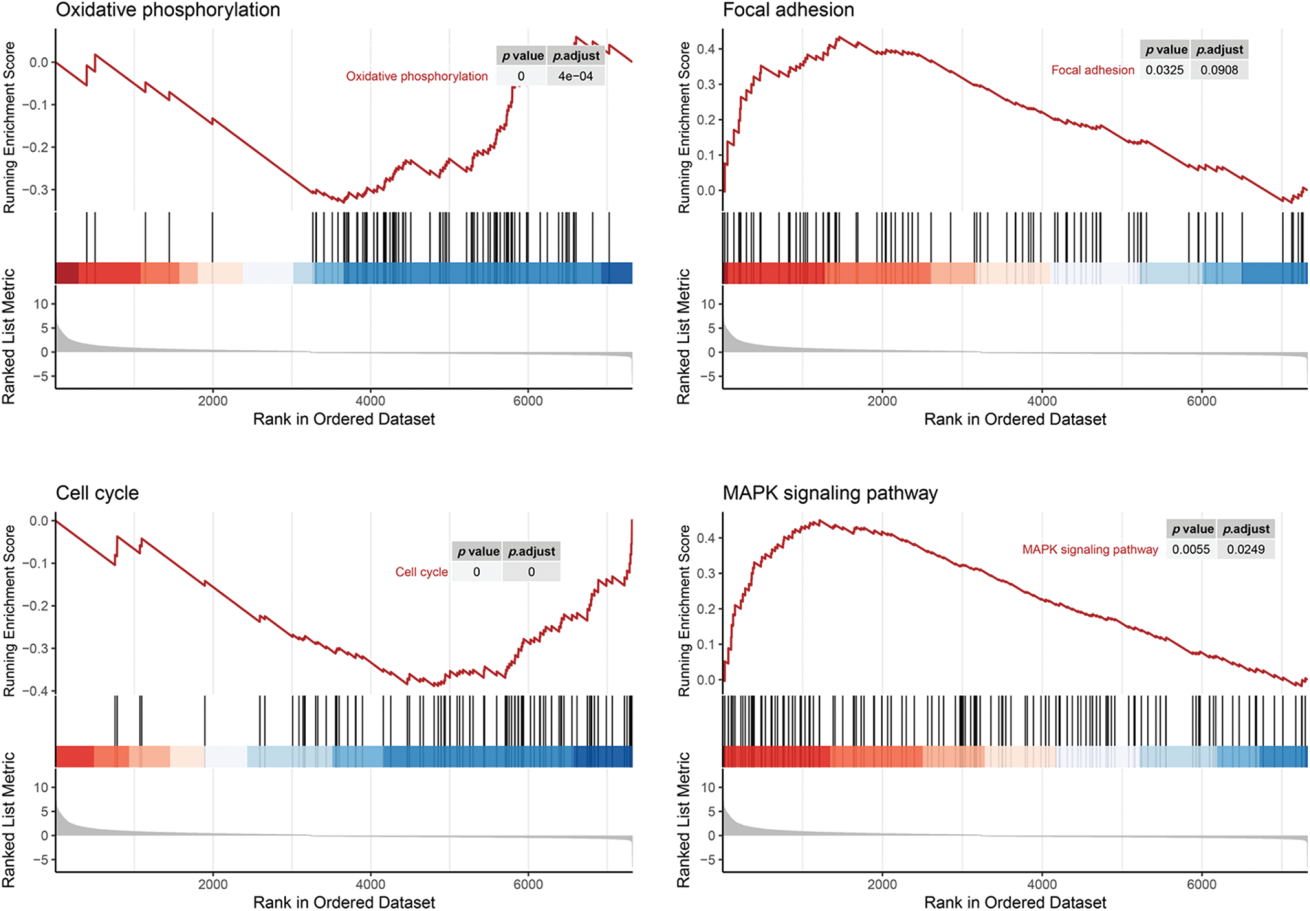
Gene set enrichment analysis of RNA-seq data and KEGG database reveals AIM2’s correlation with oxidative phosphorylation, focal adhesion, cell cycle, and the MAPK signaling pathway.

**Figure 4:**
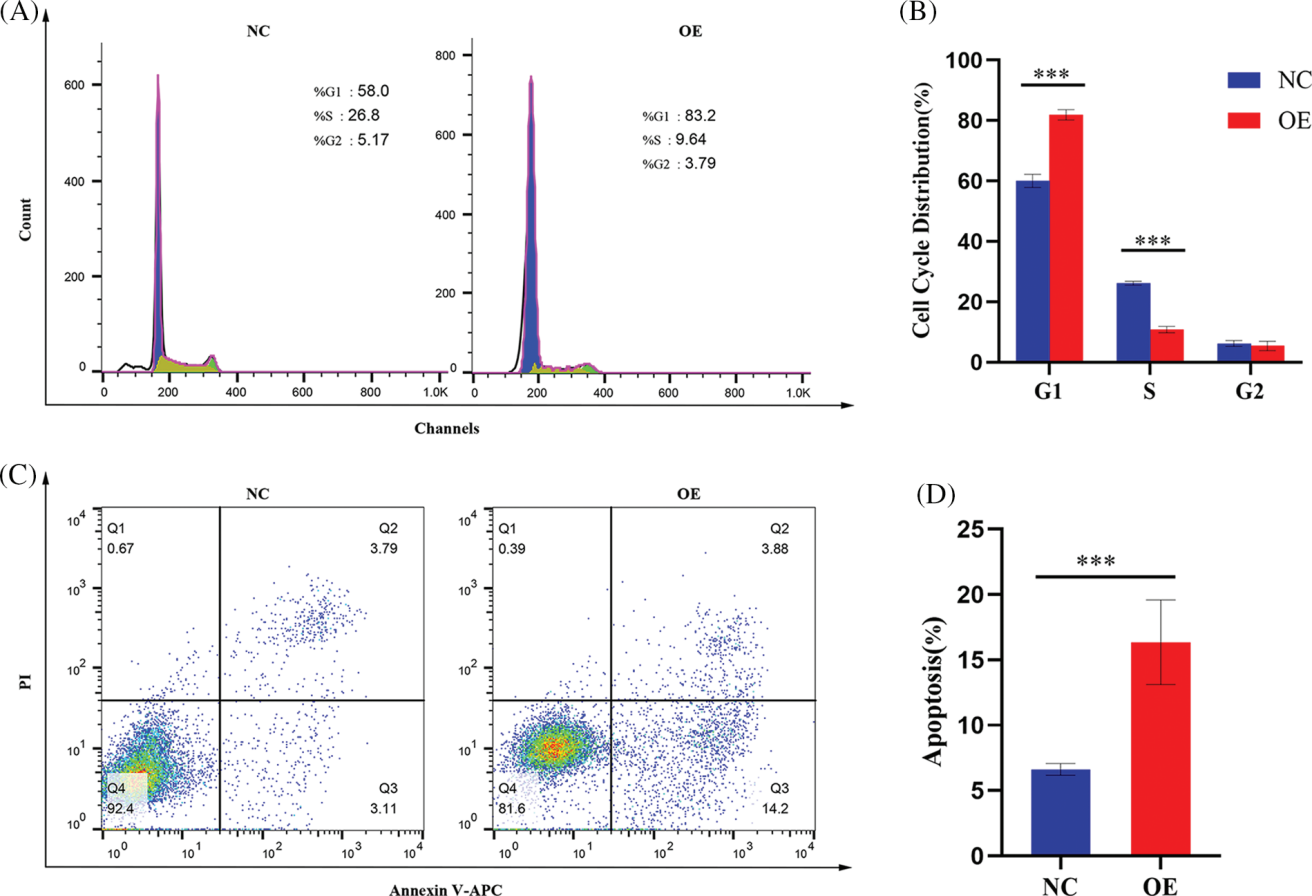
AIM2 inhibits G1/S transition and induces cell apoptosis in CRC. Flow cytometry analysis reveals (A, B) alterations in cell cycle distribution and (C, D) changes in cell apoptosis rate. Error bars represent SD (n = 3). NC: negative control; OE: AIM2 overexpression. ****p < 0.001.

Since GSEA revealed a link between AIM2 expression and the cell cycle (Fig. 3), we further investigated AIM2’s influence on CRC cell cycle progression using flow cytometry. Our results revealed an augmented proportion of cells in the G1 phase but a significantly attenuated proportion of cells in the S phase in the AIM2 OE group than in the NC group (*p* < 0.001, Figs. 4A and 4B). These findings underscored the impeding effect of AIM2 overexpression on G1/S transition in CRC cells. Subsequently, we further performed experiments to evaluate whether AIM2 affects cell apoptosis in human CRC cells. Indeed, flow cytometry analysis revealed a markedly enhanced apoptosis rate in the OE group than in the NC group (16.35% ± 3.24% *vs*. 6.62% ± 0.45%; *p* < 0.001, Figs. 4C and 4D). All these demonstrated that AIM2 arrests cell cycle and prompts apoptosis in human CRC cells.

### AIM2 regulates P38MAPK signaling pathway in CRC cells

**Figure 5:**
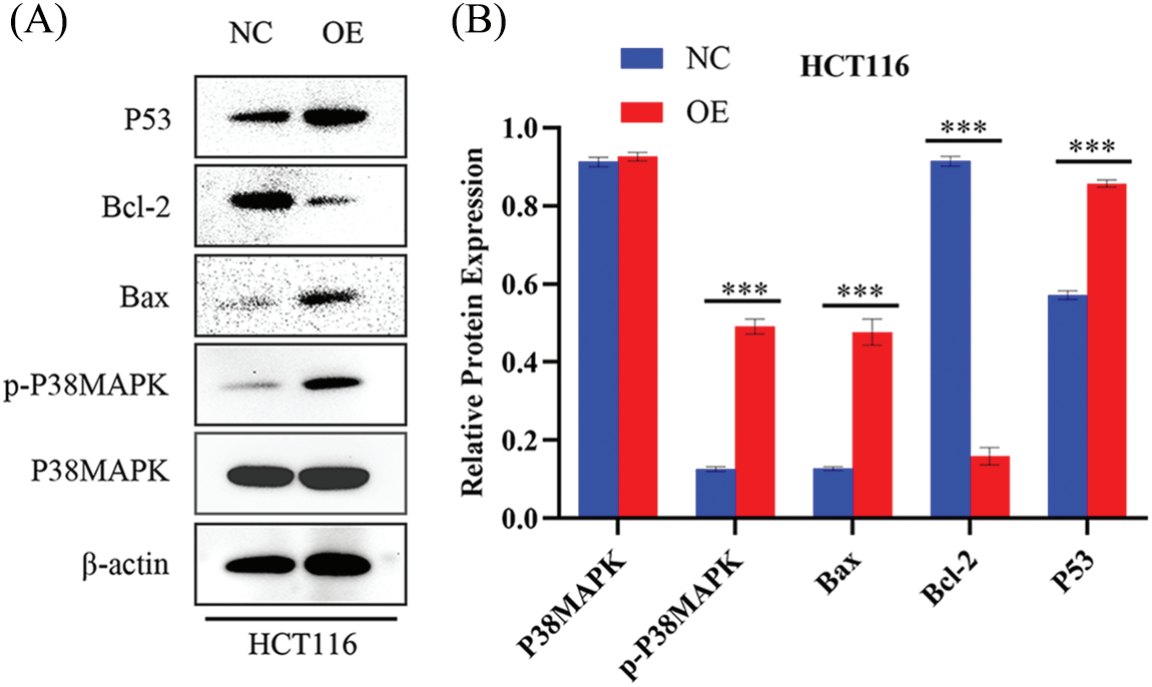
AIM2’s effect on P38MAPK signaling pathway activation in CRC cells (A, B) Western blot analysis quantifying P38MAPK, p-P38MAPK, Bax, Bcl-2, and P53 levels in HCT116 cells transfected with NC or OE. Error bars represent SD (n = 3). NC: negative control; OE: AIM2 overexpression. ***p < 0.001.

**Figure 6:**
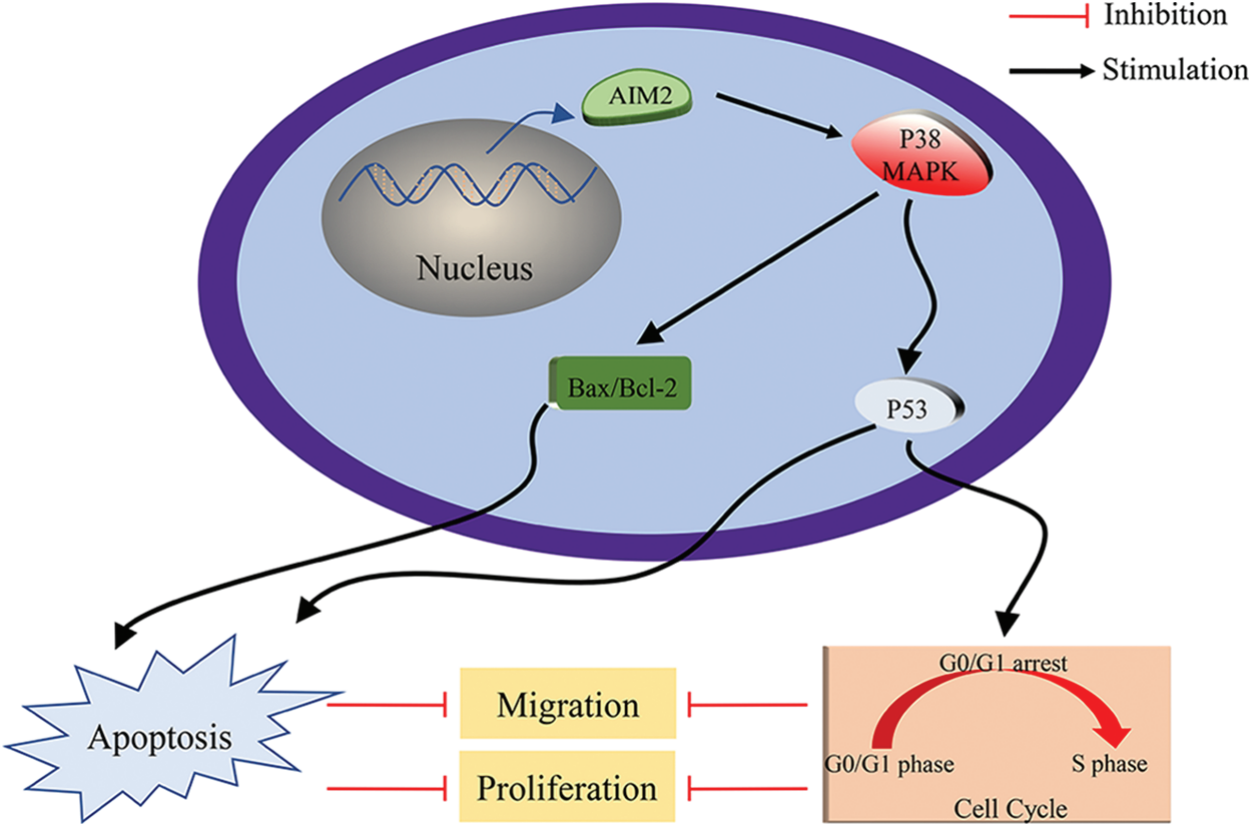
Schematic Depiction of AIM2’s role in modulating CRC cell biological behaviors via P38MAPK signaling pathway.

While our preceding findings have unveiled AIM2’s role in governing CRC cell proliferation, migration, cell cycle, and apoptosis, the potential mechanism remains enigmatic. To illustrate the mechanism of AIM2’s involvement in CRC development and progression, we performed RNA-Seq on cells in the two groups and performed GSEA analysis. Remarkably, our bioinformatics analyses indicated a connection between AIM2 expressions and several cancer-related processes and pathways, such as oxidative phosphorylation, focal adhesion, cell cycle, and the MAPK signaling pathway (Fig. 3). Especially, the GSEA of AIM2 expression showed a strong association with the MAPK signaling pathway (Fig. 3). Given the pivotal functions of the MAPK pathway and its regulators in steering CRC development and progression, we focused on P38MAPK, one of four well-known MAPKs that are intricately linked with cell proliferation, cell cycle modulation, and apoptosis. Western blot analyses revealed that compared to the NC group, phosphorylated P38MAPK (p-P38MAPK) level was significantly increased in the OE group (*p* < 0.001, Figs. 5A and 5B). Moreover, we confirmed the involvement of P38MAPK in AIM2-mediated CRC cell cycle arrest and apoptosis. Further scrutiny of P53, a well-established marker intricately linked to cancer cell cycle progression and apoptosis, reaffirmed our conclusions from flow cytometry analysis. Western blot data suggested that AIM2 overexpression markedly increased p-P38MAPK and P53 levels (*p* < 0.001, Figs. 5A and 5B). Western blot analysis of apoptosis-related proteins found that AIM2 overexpression was associated with augmented p-P38MAPK and Bax levels but reduced Bcl-2 expression level (*p* < 0.001, Figs. 5A and 5B). Altogether, these findings collectively suggest a plausible mechanism wherein AIM2 potentially curbs proliferation and promotes apoptosis in CRC cells via activating P38MAPK signaling pathway (Fig. 6).

## Discussion

The profound impact of CRC on mortality and prognosis underpins the urgency to unearth novel biomarkers tied to its onset and progression. The emerging role of AIM2 in the pathogenesis and development of various cancers, has garnered attention [11–17]. For instance, AIM2 mRNA levels are significantly reduced in prostate adenocarcinoma tissue, possibly linked to interferon signaling pathway activation [15], while they are upregulated in primary and metastatic cutaneous squamous cell carcinoma cell lines, regulating their growth and invasion [11]. Furthermore, AIM2 protein has exhibited potential in suppressing breast cancer cell proliferation and inducing cell apoptosis via inhibiting nuclear factor kappa-B activity [14]. Our previous study has reported AIM2 downregulation in CRC cell tissues and lines, indicating its promise as a diagnostic and prognostic biomarker similar to serum carcinoembryonic antigen in CRC [6]. However, despite these initial insights, our understanding of AIM2’s exact biological functions and molecular mechanisms in CRC remains incomplete, warranting further exploration.

In our present study, we re-evaluated AIM2 expression in six human CRC cells using Western blot. The findings unraveled a relatively lower AIM2 level in these cell lines, with HCT116 cells exhibiting the lowest AIM2 levels. Interestingly, HCT116, renowned for its high invasiveness, serves as a suitable model for probing tumor behavior due to its highly aggressive nature and *in vitro* motility [21–23]. Thus, we transduced an AIM2 OE lentiviral vector into HCT116 cells to overexpress AIM2, thereby facilitating an exploration of AIM2’s influence on CRC’s biological functions. Subsequently, we conducted RNA-Seq and GSEA, which suggested that AIM2 expression was associated with some cancer-related processes and pathways, including oxidative phosphorylation, focal adhesion, cell cycle, and MAPK signaling pathways. Subsequent functional assays exposed AIM2’s capacity to repress CRC cell proliferation, migration, and G1/S transition and induce apoptosis. Collectively, these findings implied a pivotal role of AIM2 in CRC occurrence and progression.

Furthermore, we explored the possible molecular mechanism underlying AIM2’s involvement in CRC development and progression. Based on RNA-Seq data and GSEA findings that AIM2 level was correlated with cell cycle and MAPK signaling pathway, we posited that P38MAPK is pivotal in governing CRC cell proliferation, cycle progression, and apoptosis [24–26]. Emerging research highlights its role in arresting the cell cycle at G1/S or G2/M checkpoints and activating P53 phosphorylation [24,27,28]. We found that AIM2 enforces G1/S phase cell cycle arrest by upregulating p-P38MAPK and P53. P53, downstream of P38MAPK, exerts a masterful control over cell cycle progression and orchestrates transitions in both G1 to S and G2 to M phases [29–31].

Apoptosis, the programmed cell death, holds paramount significance in eliminating cancer cells from the body [32,33]. Interestingly, P38MAPK is also known to be implicated in apoptosis, with previous research suggesting that the activation of P38MAPK could indirectly regulate pro-apoptotic Bax and anti-apoptotic Bcl-2 expression [34–41]. In addition, the relationship between P38MAPK and P53 is noteworthy, as P53, another downstream effector of P38MAPK, also induces apoptosis [31,42]. Interestingly, our study suggested that AIM2 induces cell apoptosis by upregulating p-P38MAPK, P53, and Bax while downregulating Bcl-2. Based on the findings of our study and other recent research, we speculated that the role of AIM2 to arrest the cell cycle and promote apoptosis might be mediated by P38MAPK. Although the precise molecular mechanisms linking AIM2 and P38MAPK warrant further investigation, our findings revealed that AIM2 overexpression suppresses CRC cell proliferation and migration via activating P38MAPK, which further prompts CRC cell cycle arrest and apoptosis.

The study bears certain potential limitations. Firstly, only HCT116 cells were chosen for some functional studies. Experiments with multiple cell lines should be carried out to verify the consistency of our findings. Secondly, experiments involving the knockdown of AIM2 in CRC cells were not performed. Thirdly, cell invasion is a special kind of cell migration, which is closely related to cell migration, both of which are common biological behaviors of tumors. The inclusion of invasion experiments could broaden insights into AIM2’s impact on CRC cell metastatic potential. Moreover, *in vivo* investigations of AIM2’s antitumor effects on CRC cells remain pivotal and merit future exploration.

Overall, our study has partly illuminated AIM2’s biological function in CRC cells. Our findings unveiled that AIM2 protein expression is notably diminished in CRC cells. Moreover, we unraveled its vital role in regulating cell proliferation and migration, inducing cell cycle arrest, and promoting cell apoptosis in CRC. Notably, we propose P38MAPK as a tumor suppressor regulating the effects of AIM2 on CRC. These findings establish a theoretical basis for recognizing AIM2 as an innovative promising therapeutic target for CRC.

## Data Availability

Data from the article are available upon request from the corresponding authors (S.G.H. and H.S.B.).
